# The Rho GTPase RAC1 in Osteoblasts Controls Their Function

**DOI:** 10.3390/ijms21020385

**Published:** 2020-01-08

**Authors:** Katrin Huck, Carla Sens, Carina Wuerfel, Caren Zoeller, Inaam A. Nakchbandi

**Affiliations:** 1 Institute of Immunology, University of Heidelberg, 69120 Heidelberg, Germany; katrin.huck@data-konzept.de (K.H.); carla.sens@daad-alumni.de (C.S.); carinat8@gmx.de (C.W.); caren.zoeller@immu.uni-heidelberg.de (C.Z.); 2 Max-Planck Institute for Medical Research, 69120 Heidelberg, and for Biochemistry, 82152 Martinsried, Germany

**Keywords:** preosteoblast, osteoblast, differentiation, Rac1, Rho GTPase, PTH, parathyroid hormone, integrin, ERK, AKT, fibronectin, EDA fibronectin, EDB fibronectin, anabolic

## Abstract

The regulation of the differentiation of the bone-forming cells, the osteoblasts, is complex. Many signaling pathways converge on the master regulator of osteoblast differentiation Runx2. The role of molecules that integrate several signaling pathways such as the Rho GTPases need to be better understood. We, therefore, asked at which stage Rac1, one of the Rho GTPase, is needed for osteoblast differentiation and whether it is involved in two pathways, the anabolic response to parathyroid hormone and the stimulatory effect of fibronectin isoforms on integrins. Genetic deletion of Rac1 in preosteoblasts using the osterix promoter diminished osteoblast differentiation in vitro. This effect was however similar to the presence of the promoter by itself. We, therefore, applied a Rac1 inhibitor and confirmed a decrease in differentiation. *In vivo*, Rac1 deletion using the osterix promoter decreased bone mineral density as well as histomorphometric measures of osteoblast function. In contrast, deleting Rac1 in differentiating osteoblasts using the collagen α1(I) promoter had no effects. We then evaluated whether intermittent parathyroid hormone (PTH) was able to affect bone mineral density in the absence of Rac1 in preosteoblasts. The increase in bone mineral density was similar in control animals and in mice in which Rac1 was deleted using the osterix promoter. Furthermore, stimulation of integrin by integrin isoforms was able to enhance osteoblast differentiation, despite the deletion of Rac1. In summary, Rac1 in preosteoblasts is required for normal osteoblast function and bone density, but it is neither needed for PTH-mediated anabolic effects nor for integrin-mediated enhancement of differentiation.

## 1. Introduction

Bone health is dependent on a balance in its turnover consisting of continuous bone resorption by osteoclasts to remove microfractures and other defects and formation by osteoblasts to restore bone. Increasing interest is being paid to the formation of new bone. Several pathways have been identified and characterized in osteoblast formation and differentiation. Bone morphogenetic proteins (BMPs) [1], fibroblast growth factors (FGFs) [2], insulin-like growth factor-I (IGFs) [3], parathyroid hormone (PTH) [4], wnt-β-catenin [5] and integrin-mediated signaling [6], all converge on a master regulator of osteoblast differentiation called Runx2 [7,8].

Three signaling pathways seem to dominate in mediating osteoblast differentiation. Parathyroid hormone, integrin signaling and canonical wnt signaling. Parathyroid hormone (PTH) is a hormone that regulates calcium in the bloodstream. It binds to the type I PTH/PTHrP receptor, a G-protein coupled receptor (GPCR) on the surface of osteoblasts leading to signaling through the PKA and PKC pathways and activation of differentiation. Unfortunately, this also leads to an indirect activation of the osteoclasts to enhance resorption by various mechanisms including cytokine release [9,10,11]. Its intermittent administration, on the other hand, enhances bone formation and has been used therapeutically [4].

Activating the integrin signaling pathway can also enhance osteoblast differentiation [6,12]. Integrins are receptors consisting of each of dimers of a α and a β subunit. They are located on the cell surface and transmit signals and other cues from the extracellular surrounding to affect cell behavior [13]. Mesenchymal stromal cells in the bone marrow can differentiate into osteoblasts, and α4β1 and α5β1 integrins appear to be involved in mesenchymal cell differentiation [14]. These integrins bind to different extracellular matrix proteins: α4β1 binds to an isoform of fibronectin called EDA-fibronectin and α5β1 is the classical fibronectin receptor [13,15]. Furthermore, osteoblast differentiation is enhanced by engaging αvβ3 integrin [6,12].

The canonical wnt signaling pathway has also been studied [16], Indeed, studies on the canonical wnt signaling sclerostin led to the development of a therapeutic agent directed against the inhibitor sclerostin for the treatment of osteoporosis that was recently approved for use in patients in the US [17].

Crosstalk between these pathways has been proposed by different groups: through the convergence of PTH and integrin signaling pathways on Runx2 [8], or through indirect interactions in mesenchymal stromal cells between integrin and wnt pathways [5]. Furthermore, PTH actions may be partially mediated by effects on the wnt signaling pathway [18], but much remains to be clarified. Molecular candidates for crosstalk between different signaling pathways are the Rho GTPases, which consist of several members [19,20]. One of them is Rac1 (ras-related C3 botulinum toxin substrate 1), which is a small key effector molecule that is ubiquitously expressed. Rac1 transmits signals originating from the integrin-signaling pathway [21], and the wnt signaling pathway [22]. It, therefore, is well situated to integrate signals involved in osteoblast differentiation. Published work suggests that the deletion of Rac1 results in diminished osteoblast differentiation, but further characterization lacks [23,24].

The aim of this work, therefore, is to characterize the role of Rac1 in osteoblasts and in the integration of signaling pathways involved in osteoblast differentiation.

## 2. Results

### 2.1. Rac1 Is Expressed in Osteoblasts and Can Be Genetically Deleted

**Figure 1:**
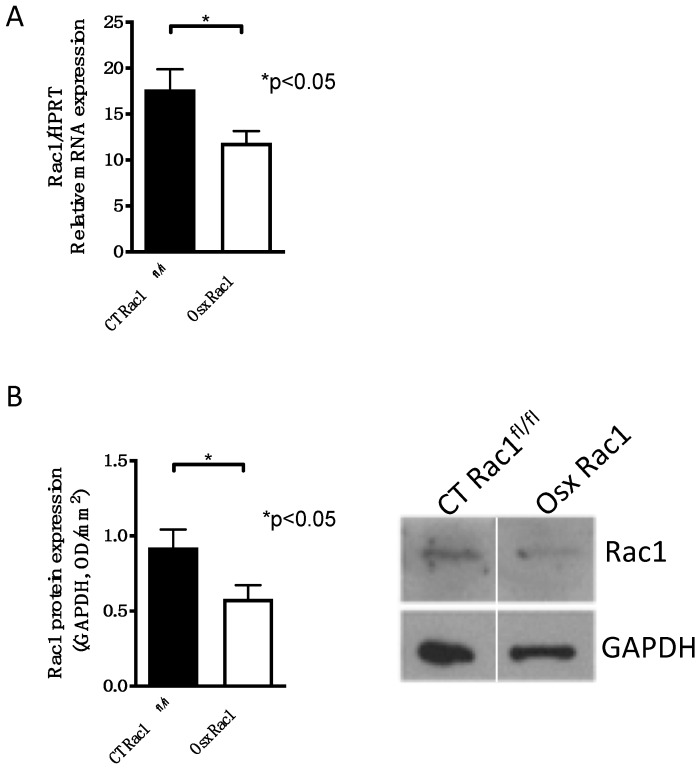
Successful deletion of Rac1 in osteoblasts using osterix to drive Cre recombinase expression. (**A**) Rac1 mRNA expression is diminished in primary newborn calvarial osteoblasts isolated from Osx Rac1 animals compared to cells from littermate controls (CT Rac1^fl/fl^). Rac1 was genetically deleted using osterix to drive Cre recombinase expression in mice homozygous for the floxed Rac1 gene (Osterix-Cre/+_Rac1^fl/fl^: Osx Rac1). The mice were not exposed to doxycycline at any time pre- or postnatally. *n* = 10/10. Rac1 mRNA was adjusted to the housekeeping gene HPRT and the values compared by a *t*-test. (**B**) Rac1 protein is diminished in primary calvarial newborn osteoblasts from Osx Rac1 mice compared to controls. *n* = 15/13. The protein was adjusted to the housekeeping protein GAPDH. The values were then compared between both groups using a *t*-test.

A complete knockout of Rac1 leads to the death of embryos during development, because of its ubiquitous expression and involvement in several cellular processes [25]. Since we aimed to evaluate the role of Rac1 in osteoblasts, we chose to delete it genetically. Mice carrying the promoter osterix to drive cre recombinase expression (Osx) were mated over two generations with Rac1 floxed/floxed mice (Rac1^fl/fl^) in order to generate mice that carry Cre (under the control of the Osx promoter) with Rac1^fl/fl^ (Osx Rac1). Osx and hence Cre recombinase expression can be repressed using doxycycline during pregnancy and postnatally. For the study of newborn calvarial osteoblasts, pregnant mothers did not receive any doxycycline. Using the osterix promoter to drive Cre recombinase expression in primary osteoblasts isolated from newborn calvaria, we found that Rac1 mRNA, as well as protein expression, was detected in primary osteoblasts in controls (CT Rac1^fl/fl^) and diminished in cells isolated from Osx Rac1 animals (Figure 1A,B and Supplementary Data A).

### 2.2. Inhibition of Rac1 Suppresses Osteoblast Differentiation

**Figure 2:**
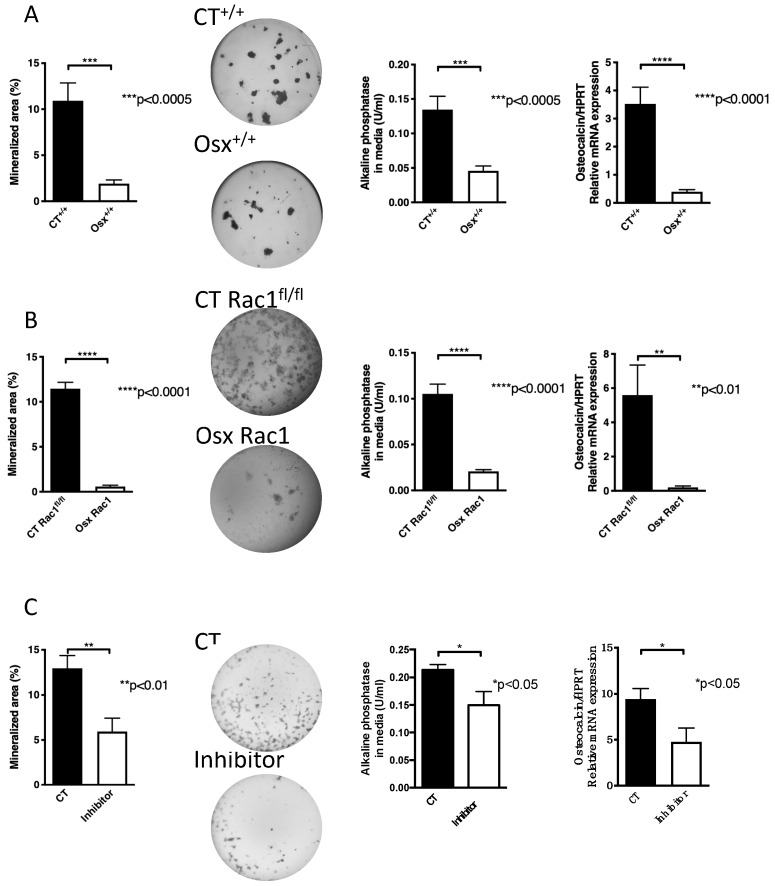
Inhibition of Rac1 affects osteoblasts. (**A**) The expression of osterix to drive Cre recombinase without deletion of Rac1 (Osx^+/+^) in newborn calvarial osteoblasts suppresses their differentiation in vitro compared to littermate controls not carrying any gene (CT^+/+^). Newborn calvarial osteoblasts were isolated and cultured for 2-3 weeks in the mineralizing condition in the presence of vitamin C, β-glycerophosphate, and dexamethasone added fresh with each medium change (3 times/week). At the end, the wells are stained with von Kossa, the media evaluated for alkaline phosphatase, and mRNA from the cells evaluated for osteocalcin mRNA expression adjusted to HPRT. The mice were not exposed to doxycycline at any time pre- or postnatally. *n* = 19/16, 20/20, and 17/20. (**B**) Deletion of Rac1 using osterix-driven Cre recombinase (Osx Rac1) leads to diminished mineralization, alkaline phosphatase and osteocalcin/HPRT compared to littermate controls (CT Rac1^fl/fl^). The mice were not exposed to doxycycline at any time pre- or postnatally. *n* = 12/19, 32/32, and 3/5. (**C**) Using a chemical inhibitor of Rac1 (EHT1864) added with each medium change at a final concentration of 5 μM suppressed differentiation of newborn calvarial osteoblasts as evidenced by suppressed nodule formation in cultures stained with von Kossa, suppressed alkaline phosphatase in the conditioned media and lower levels of osteocalcin/HPRT mRNA expression compared to control cells treated with phosphate-buffered saline (PBS) containing dimethylsulfoxide (DMSO) at the same concentration as the inhibitor. *n* = 10/15, 19/19, 12/12. The values were compared between every two groups in all subpanels using *t*-tests.

Since we aimed to evaluate the role of Rac1 in preosteoblasts we decided to use Osx to drive Cre expression in order to delete Rac1. At first, we examined whether Osx-Cre expression by itself without deleting any gene (Osx^+/+^) has any effect on osteoblast differentiation in vitro. Newborn calvarial osteoblasts were isolated and cultured for 2–3 weeks in mineralizing condition to perform what is known as the nodule formation assay in the absence of doxycycline-mediated cre-recombinase expression prenatally. Wells were then stained with von Kossa and the mineralized area was photographed and quantified. We found that expression of Cre under the control of the osterix promoter (Osx^+/+^) diminished nodule formation compared to littermate controls (CT^+/+^) that do not express Osx-Cre. Furthermore, alkaline phosphatase in the conditioned media at the end of the experiment was diminished as was osteocalcin mRNA expression in Osx^+/+^ newborn osteoblasts compared to littermate CT^+/+^ (Figure 2A). Since Cre recombinase was knocked into the mouse genome next to the osterix promoter, the effect on osteoblasts could be either due to a decrease in endogenous osterix expression or to Cre expression by the osterix promoter. Evaluation of osterix mRNA expression by another group, however, failed to show a difference [26].

Similarly, the deletion of Rac1 using Osx-Cre (Osx Rac1) also showed a decrease in differentiation compared to littermate control mice carrying Rac1^fl/fl^ without Osx and labeled CT Rac1^fl/fl^ (nodule formation, alkaline phosphatase and osteocalcin: Figure 2B). The degree of reduction in Osx Rac1 seemed larger than in Osx^+/+^ (Nodule formation was reduced by 95% in Osx Rac1 vs. 82% in Osx^+/+^ when each was compared to its control). These findings, therefore, suggest an important role for Rac1 in osteoblasts.

To validate the finding that Rac1 affects osteoblast differentiation in vitro irrespective of the presence or absence of Osx, we used a pharmacological inhibitor of Rac1. We first tested the effect of adding the inhibitor at increasing concentrations at every medium change to osteoblasts in mineralizing conditions. We found a dose-dependent decrease in nodule formation with higher concentrations of the inhibitor as evaluated by ANOVA. We used the lowest dose that seemed to affect nodule formation, albeit not significantly (Supplementary Figure S1) and found using a larger number of replicates a decrease in the three differentiation measures: nodule formation, alkaline phosphatase in the media, and osteocalcin mRNA expression (Figure 2C).

Taken together, these data show that osteoblast differentiation diminishes in the absence of Rac1.

### 2.3. Deletion of Rac1 Affects Bone Mineral Density and Osteoblast Function In Vivo

**Figure 3:**
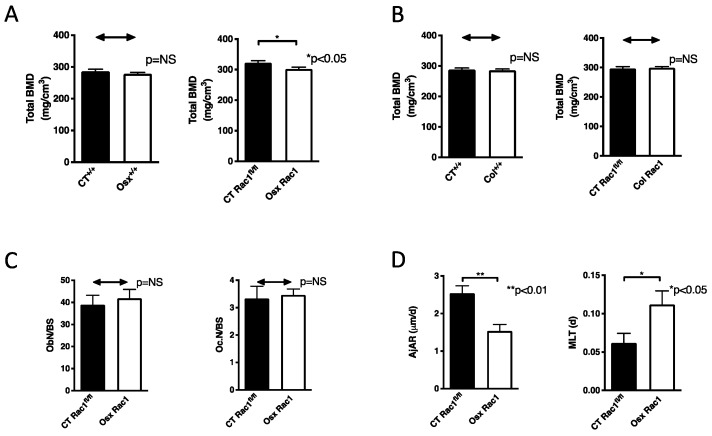
Bone mineral density and osteoblast differentiation are decreased in the absence of Rac1 in preosteoblasts. (**A**) Bone mineral density (BMD) in the presence of osterix-Cre in wildtype mice (Osx^+/+^) was not affected compared to wildtype littermate control mice (CT^+/+^), but deletion of Rac1 using osterix to drive Cre expression (Osx Rac1) diminished bone mineral density compared to littermate controls with the genotype Rac1^fl/fl^ (CT Rac1^fl/fl^). *n* = 27/37 and 20/19. (**B**) Bone mineral density in the presence of collagen α1(I)-Cre in wildtype mice (col^+/+^) was not affected compared to wildtype littermate control mice (CT^+/+^). Similarly, the deletion of Rac1 using collagen α1(I) to drive Cre expression (Col Rac1) did not affect bone mineral density compared to littermate controls with the genotype Rac1^fl/fl^ (CT Rac1^fl/fl^). *n* = 18/18 and 18/23. (**C**) Deletion of Rac1 using Osx-Cre did not affect the number of osteoblasts (Ob.N) or osteoclasts (Oc.N) when adjusted to bone surface (BS). Data were obtained using static histomorphometry of tibia sections after excluding the primary spongiosa for a width of 150 μm. The bone was evaluated along the longitudinal axis for a total length of 1.5 mm starting from the exclusion line distally. *n* = 10/5. (**D**) Osteoblast function was diminished in Osx Rac1 mice compared to littermate controls. The adjusted apposition rate (AjAR) reflects the function of a group of osteoblasts, which is diminished, while mineralization lag time (MLT) represents the time needed for the osteoblasts to mineralize the matrix. The function is diminished if MLT is prolonged. *n* = 10/5. All studies were performed without doxycycline treatment and repression of Cre recombinase expression. Bone mineral density was measured in 3 weeks old mice. The values were compared between every two groups in all subpanels using *t*-tests.

Despite the more pronounced decrease in osteoblast differentiation in Osx Rac1 vs. Osx^+/+^ compared to their controls, the decrease in osteoblast differentiation in vitro using Osx-Cre by itself raised the possibility that Osx-Cre cannot be used to evaluate effects of loss of Rac1 in vivo on bones or that Osx Rac1 conditional knockout (cKO) mice need to be compared to Osx^+/+^ and not to Rac1^fl/fl^ mice. We, therefore, evaluated bone mineral density of the femur in Osx^+/+^ mice compared to their littermate controls in the absence of postnatal cre repression (no doxycycline was given). No effect of Osx expression attached to cre in wildtype mice on bone density could be detected. In contrast, the deletion of Rac1 in preosteoblast (Osx Rac1) led to a significant decrease in bone mineral density compared to their littermate controls (CT^+/+^). This decrease, however, was small (Figure 3A). Based on the lack of difference between Osx^+/+^ and CT ^+/+^, it seemed that the use of Osx^+/+^ mice as a control for Osx Rac1 was not required. We then sought to determine whether deletion of Rac1 for one week only is enough to affect bone mineral density, we treated the pregnant mice as well as the mothers during nursing with doxycycline to repress osterix expression for a total of two weeks postnatally, and stopped doxycycline for 1 week before killing the mice. No change in bone mineral density could be detected (Supplementary Figure S2). This repression of osterix and hence Cre recombinase expression cannot be used to study the effect of Rac1 in preosteoblasts on bone mineral density.

To find out whether the decrease in bone mineral density in Osx Rac1 is limited to the deletion of Rac1 in preosteoblasts or whether it also takes place in differentiating osteoblasts, we used the collagen α1(I) promoter to drive Cre recombinase expression (Col). By itself, the expression of collagen α1(I)-Cre in wildtype mice (Col^+/+^) did not affect bone mineral density, as was the case using Osx-Cre by itself. Deletion of Rac1 using the collagen α1(I) promoter to drive cre expression did not result in any change in bone mineral density (Figure 3B). Thus, Rac1 expression in preosteoblasts, but not in differentiating osteoblasts, is required for maintenance of bone health.

In order to determine the reason for the decrease in bone density, we performed static and dynamic histomorphometry on the tibia after the exclusion of the primary spongiosa with a thickness of 150 μm. The region evaluated included cortical and trabecular bone along the longitudinal axis of the tibia for a total length of 1.5 mm starting from the exclusion line. The deletion of Rac1 using Osx-Cre did not affect the total number of osteoblasts or osteoclasts (when corrected to the bone surface) (Figure 3C and Supplementary Figure S3A). The osteoblasts exhibited poor function as evidenced by a decrease in osteoid thickness, which represents the matrix laid down by the osteoblasts before it is mineralized (Supplementary Figure S3B). While mineralization surface (MS) was also diminished in Osx Rac1 animals, this was no longer the case when adjusted to bone surface (Supplementary Figure S3C). Together with the change in osteoid thickness these data suggested a change in osteoblast function in vivo. Indeed, two calculated values representing osteoblast function: adjusted apposition rate (AjAR), which reflects the activity of a team of osteoblasts (It takes into account the mineralization speed of the osteoid expressed a mineral apposition rate (MAR), mineralizing surface (MS) as well as osteoid surface (OS) and is calculated as follows: AjAR = MAR × MS/OS, μm/day), and mineralization lag time (MLT), which represents the time required for the osteoblasts to mineralize the newly laid down matrix (It takes into account osteoid thickness (O.Th) which represents the thickness of the newly laid down, not yet mineralized matrix and AjAR and is calculated as follows: MLT = O.Th/AjAR, days). AjAr was diminished in Osx Rac1 suggesting impaired osteoblastic activity and MLT was increased, which is consistent with a delay in the mineralization of the osteoid by osteoblasts (Figure 3D). Thus, depletion of Rac1 in preosteoblasts diminishes osteoblast function leading to decreased bone mineral density.

### 2.4. Deletion of Rac1 in Preosteoblasts Does Not Prevent the Response to PTH

It was shown during embryonic development that accumulation of β-catenin in the nucleus and hence canonical wnt signaling requires Rac1 activation [22]. Furthermore, in vitro Rac1 seems to mediate wnt signal effects [24]. Since intermittent parathyroid hormone stimulates bone formation and a major pathway for bone formation is driven by wnt signaling we hypothesized that the deletion of Rac1 in preosteoblasts will prevent the increase in bone formation in response to PTH [16,27].

**Figure 4:**
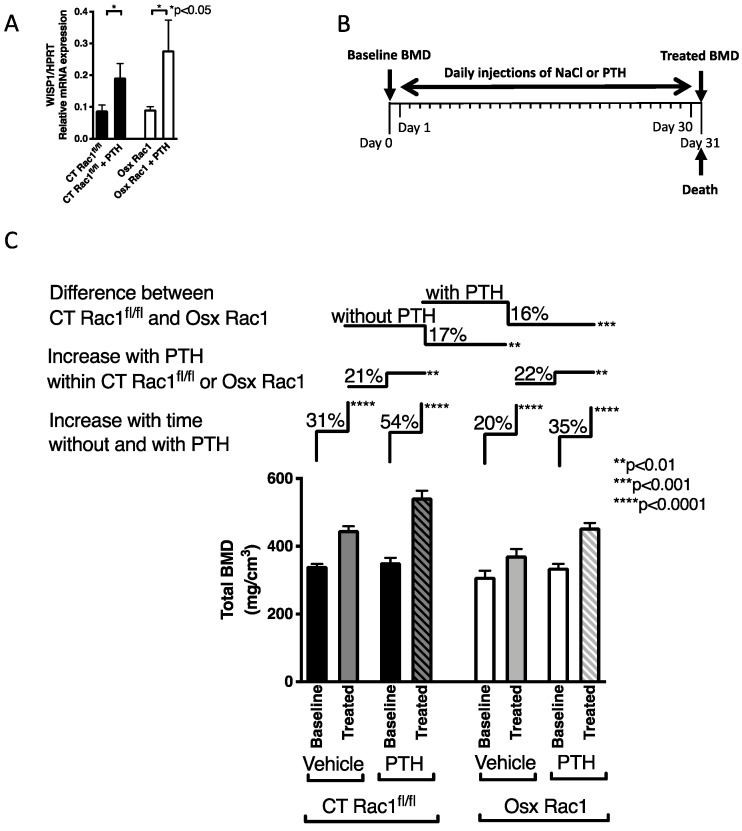
Rac1 is not required for mediating parathyroid hormone (PTH) anabolic effects in bone. (**A**) PTH treatment of freshly isolated osteoblasts in vitro increases WISP1 in both CT Rac1^fl/fl^ and Osx Rac1. *N* = 5/4/5/3. Data were analyzed by ANOVA (*p* < 0.05) followed by unpaired *t*-tests. (**B**) Study design for PTH injection: at the age of 4 weeks, bone mineral density (BMD) was measured in CT Rac1^fl/fl^ and Osx Rac1 mice. Mice were then divided into two CT Rac1^fl/fl^ groups and two Osx Rac1 groups that had comparable bone mineral density (mean and range). In each genotype, the first group received daily vehicle injections and the second group daily PTH injections (80 μg/kg/day) for 30 days. At the end of the experiment, mice were killed, and bone mineral density measured. None of the mice was exposed to doxycycline postnatally. (**C**) Treatment with daily subcutaneous PTH injection increased bone mineral density in all mice. Bone mineral density of vehicle-treated mice increased as they got older in CT Rac1^fl/fl^ (31%), but this increase was blunted in Osx Rac1 mice (20%). PTH increased bone mineral density in CT Rac1^fl/fl^ (21%) and Osx Rac1 mice (22%). Despite starting at a lower bone mineral density, Osx Rac1 mice seemed to respond adequately to age progression and to PTH, because the difference in bone density between CT Rac1^fl/fl^ and Osx Rac1 remained constant irrespective of whether PTH was administered or not (17 and 16%). *N* = 9/9, 14/14, 14/13, 22/22. Vehicle or PTH was injected daily subcutaneously in 4 weeks old mice for 30 days. Bone mineral density was measured before and after therapy. The percentages represent the increase or decrease in bone mineral density of the second value based on the first value (for the first pair: percentage change = (treated-baseline)/baseline). For statistical analysis, ANOVA was performed and found to be statistically significant with a *p* < 0.0001 allowing further comparisons. Each pair was evaluated either in paired *t*-tests (for the paired comparisons of baseline to treated group next to the label: “Increase with time without and with PTH”) or unpaired *t*-test (for the comparisons of treated groups together next to the labels: “Increase with PTH within CT Rac1^fl/fl^ or Osx Rac1” as well as “Difference between CT Rac1^fl/fl^ and Osx Rac1”).

We first evaluated the response of osteoblasts to PTH in vitro. Freshly isolated newborn calvarial osteoblasts were exposed to PTH for 24 h and mRNA was isolated (Figure 4A). We evaluated wnt1-inducible signaling pathway protein-1 (WISP-1), also known as CCN4 and which is a β-catenin regulated gene that is activated by wnt-1 [28]. Both in CT and Osx Rac1 cells, WISP-1 increased with PTH treatment. This suggested that at least for this gene Rac1 is not required.

We, therefore, proceeded with in vivo experiments (Figure 4B). Our aim was to evaluate whether intermittent PTH injections can mount an anabolic response increasing bone mineral density in the absence of Rac1 in preosteoblasts (Osx Rac1). Since the deletion of Rac1 for only 1 week was not enough to affect bone mineral density, we did not use doxycycline to repress Osx-Cre (Supplementary Figure S2). As shown above, Osx^+/+^ by itself did not affect bone mineral density compared to CT^+/+^. Therefore, it seemed reasonable to use littermate controls of any genotype. Our mating scheme was therefore performed using Osx Rac1 with Rac1^fl/fl^. Thus, we only obtained two genotypes: Osx Rac1 and CT Rac1^fl/fl^. We treated both CT Rac1^fl/fl^ and Osx Rac1 mice at the age of 4 weeks with PTH daily injections (80 μg/kg/d) for 30 days and evaluated the effect on bone mineral density. As shown in Figure 4, both CT Rac1^fl/fl^ and Osx Rac1 mice increased their bone density with increasing age (by 31% and 20% for CT Rac1^fl/fl^ and Osx Rac1, respectively). Furthermore, and as expected, PTH administration over 30 days increased bone mineral density in CT Rac1^fl/fl^ mice by 54%, while in Osx Rac1 mice the increase was limited to 35%. This suggests that even though the absence of Rac1 diminishes osteoblast activity, PTH is still able to increase bone density in the absence of Rac1 in preosteoblasts. While the difference between vehicle-treated CT Rac1^fl/fl^ and Osx Rac1 mice was 17% in untreated animals, this difference remained after PTH treatment between CT Rac1^fl/fl^ and Osx Rac1 at 16%. Interestingly, the increase attributed to PTH in CT Rac1^fl/fl^ mice was 21% and in Osx Rac1 22%. Taken together, these data show that Rac1 is not critical for mediating PTH effects on osteoblasts in vivo.

### 2.5. Rac1 in Preosteoblasts Is Involved in Mediating Integrin Stimulation of Osteoblasts

**Figure 5:**
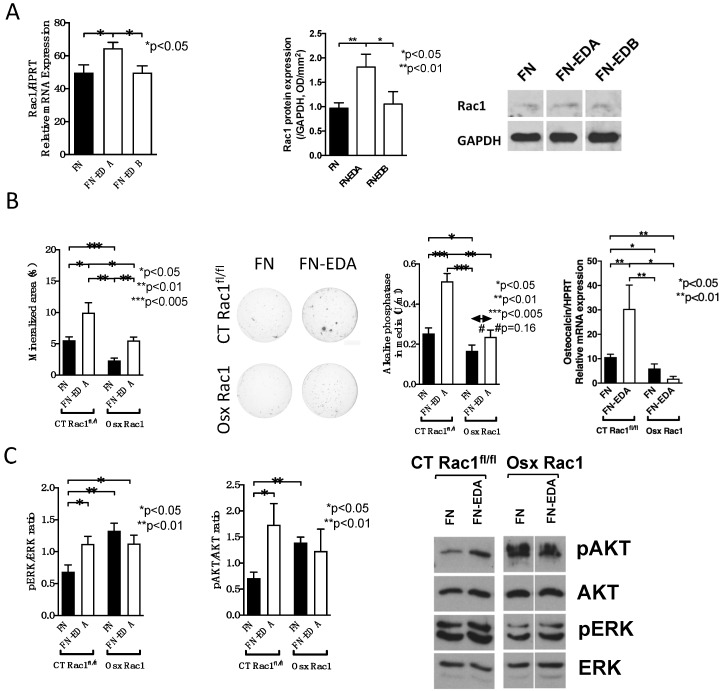
Conditional deletion of Rac1 in preosteoblasts interferes with integrin signaling. (**A**) Only transfection with fibronectin containing EDA (FN-EDA) increases Rac1 mRNA and protein expression. *n* = 8/7/7 and 10/9/6. Rac1 mRNA was adjusted to HPRT and Rac1 protein to GAPDH. (**B**) Transfection with FN-EDA enhances mineralization in CT Rac1^fl/fl^ and in Osx Rac1 osteoblasts. Unlike CT Rac1^fl/fl^ cells, however, alkaline phosphatase in the media and osteocalcin mRNA do not respond in Osx Rac1. Newborn calvarial osteoblasts were isolated, transfected with the FN or FN-EDA construct and cultured for 2–3 weeks in mineralizing conditions in the presence of vitamin C, β-glycerophosphate, and dexamethasone added fresh with each medium change (3 times/week). At the end, the wells were stained using von Kossa stain and the media evaluated for alkaline phosphatase. Osteocalcin/HPRT mRNA was evaluated in sister wells that were not stained or in separate experiments and corrected to the housekeeping gene HPRT. Mice were not exposed to doxycycline at any time. *n* = 7/6/5/7, 6/5/5/4, 10/3/8/3. (**C**) ERK and AKT phosphorylation increased upon transfection with FN-EDA in CT Rac1^fl/fl^ cells. In Osx Rac1 the phosphorylation of both increased with FN and did not increase further with FN-EDA. Cells were transfected and left two days before collecting the cell lysate for Western blotting. *n* = 7/6/9/12, 7/7/5/7. ANOVA was performed and whenever significant, the values were compared between every two groups in all subpanels using *t*-tests.

We next sought to evaluate whether Rac1 is required for integrin-mediated osteoblast differentiation. We had shown that two isoforms of the extracellular matrix protein fibronectin, the fibronectin containing EDA (FN-EDA) as well as the fibronectin containing EDB (FN-EDB) stimulate osteoblast differentiation by binding to α4β1 and αvβ3 integrins respectively [6]. We, therefore, transfected these two constructs in wildtype osteoblasts and evaluated whether Rac1 mRNA and total Rac1 protein expression were changed. Interestingly, only FN-EDA affected Rac1 expression both at the mRNA and protein levels (Figure 5A and Supplementary Data B). For this reason, we only evaluated the role of FN-EDA in Rac1-deleted osteoblasts.

We then induced osteoblast differentiation in vitro by transfecting the control construct of fibronectin lacking EDA (FN) and the one containing EDA (FN-EDA) in CT Rac1^fl/fl^ and in Osx Rac1 newborn calvarial osteoblasts (Figure 5B). The absence of Rac1 diminished in vitro mineralization, alkaline phosphatase, and osteocalcin mRNA expression. The transfection with FN-EDA was able to enhance mineralization in CT Rac1^fl/fl^ and Osx Rac1 osteoblasts, but the increase in CT Rac1^fl/fl^ seen for alkaline phosphatase and osteocalcin could not be replicated in Osx Rac1 cells. Thus, while differentiation is improved with activation of EDA-binding integrin in Osx Rac1 it does not completely normalize.

Evaluation of phosphorylation of the downstream molecules of integrin activation ERK and AKT confirmed the increase with FN-EDA transfection in CT Rac1^fl/fl^ osteoblasts, but baseline expression was already increased in FN-transfected Osx Rac1 cells and did not increase further with FN-EDA transfection (Figure 5C and Supplementary Data C). This suggests that the deletion of Rac1 influences intracellular signaling, but no additional stimulation can be achieved with activation of FN-EDA binding α4β1 integrin.

In summary, the absence of Rac1 in newborn calvarial osteoblasts diminishes their differentiation but does not prevent the improvement that is mediated by integrin activation, even though alterations in intracellular signaling can be detected.

## 3. Discussion

**Figure 6:**
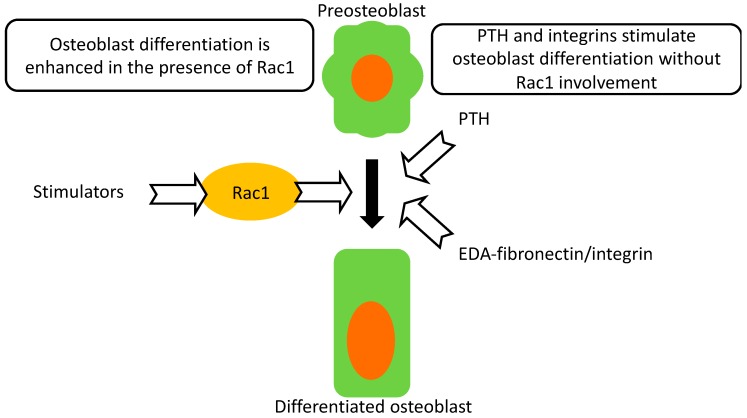
Summary. Osteoblast differentiation proceeds normally in the presence of Rac1. Rac1, however, is not required for intermittent PTH anabolic effects. EDA-fibronectin-mediated enhancement of differentiation through activation of α4β1 can also proceed in the absence of Rac1. Thus, Rac1 is a modulator of differentiation but is not an absolute requirement for differentiation to proceed.

Deletion of Rac1 in preosteoblasts, but not in differentiating osteoblasts suppresses their differentiation. This leads to a small decrease in bone mineral density as well as histologic evidence of impairment in osteoblastic function. Despite this impairment, intermittent parathyroid hormone administration is still able to mount a robust response and improve bone mineral density. Furthermore, enhanced differentiation mediated by integrin activation proceeds, despite evidence of perturbed intracellular signaling. Neither PTH- nor integrin-mediated enhanced differentiation in Osx Rac1 can reach the values found in treated control cells or mice (Figure 6).

The Rho GTPase Rac1 is required during development because its deletion is embryonic lethal [25]. It controls the actin cytoskeleton organization and is needed for lamellipodia formation, a structure that forms on the leading edges of cells to allow for movement and migration [29]. Rac1 has been implicated in transmitting signals from the matrix via integrins to prevent cell death (anoikis) [30]. In fibroblasts, the deletion of Rac1 led to the disruption of cell-fibronectin interaction [31]. Since the extracellular matrix protein fibronectin was shown to stimulate osteoblast differentiation [6,32], it can, therefore, be assumed that Rac1 is involved in osteoblast differentiation. Indeed, it was shown that Rac1 deletion in an osteoblastic cell line led to decreased proliferation and increased apoptosis and in mice led to a decrease in bone mineral density [23]. Our work shows that the role of Rac1 is limited to preosteoblasts because its deletion in differentiating osteoblasts failed to show any effect (Figure 3A,B). Furthermore, Rac1 is needed for new bone formation (Figure 3D).

Osteoblasts respond to parathyroid hormone (PTH) by diminished apoptosis [33] and increased differentiation [34]. Continuous elevation enhances bone formation and bone resorption leading to a net loss of bone such as in primary hyperparathyroidism [9,35]. If given intermittently, osteoblast differentiation is increased more so than osteoclast differentiation. One possibility is that the release of TGF-β from resorbed bone leads to the recruitment of additional osteoblastic progenitors [34]. PTH stimulates osteoblast differentiation by acting on Runx2 and possibly activating several pathways. One of them, PKA, has been implicated in wnt signaling [36]. Based on work showing that Rac1 controls β-catenin and hence activation of the wnt signaling pathway during embryonic development [22], as well as in vitro evidence that Rac1 modulates wnt signaling in osteoblasts [24], we asked whether Rac1 might affect the anabolic response to intermittent PTH. Our findings show that PTH increases bone mineral density similarly between CT Rac1^fl/fl^ and Osx Rac1 mice when evaluated relative to the starting bone mineral density. Thus, despite a lower bone density at baseline, the response to PTH in Osx Rac1 seems normal. In conclusion, PTH anabolic effects are independent of Rac1-mediated signaling.

Another important osteoblastic differentiation pathway is the one mediated by integrin activation. We had shown that osteoblast differentiation is stimulated in an autofeedback loop by two isoforms of fibronectin (FN): the one containing an extra domain A (FN-EDA) and the one containing the extra domain B (FN-EDB) [6,32]. Since only FN-EDA changes Rac1 expression, we evaluated the role of FN-EDA isoform compared to the one lacking any extra domain (FN). In the absence of Rac1, osteoblast differentiation was suppressed, but transfection with the FN-EDA construct stimulated nodule formation both in CT Rac1^fl/fl^ cells [6], and in Osx Rac1 cells (Figure 5B). This suggests that in the absence of Rac1 a seemingly robust increase in nodule formation ensues. More interesting are the changes in pERK and pAKT, which were elevated at baseline in Osx Rac1 cells and could not increase further with α4β1 integrin activation (α4β1 is the integrin activated by the presence of the EDA domain in fibronectin). Since this did not go hand in hand with an increase in differentiation, it is possible that in the absence of Rac1 other mechanisms allow the osteoblasts to proceed in their differentiation. An explanation for the increase in the signaling molecules could be that Rac1 in osteoblasts normally suppresses signaling through ERK and AKT and in its absence, an upregulation ensues. Crosstalk between Rac1 and ERK or AKT in the opposite direction to what we found has been reported. Rac1 induces integrin-mediated ERK activation [37], but ERK also can induce the activation of Rac1 [38]. Similarly, it was shown that Rac1 is located upstream of AKT and required for its activation [39], but that AKT can also act upstream of Rac1 [40]. However, both Rac1 and AKT can also act independently [41]. Thus, more work is needed to understand the intricacies of osteoblast differentiation.

Our study has some limitations. The deletion of Rac1 in the Osx Rac1 mice was limited as evidenced by the presence of a signal for Rac1 in Western blots of newborn calvarial osteoblasts. This suggests that osterix is only expressed in a limited number of the cells isolated from the calvaria and traditionally used for osteoblast assays. Therefore, other cells that express Rac1 are still present in the bone microenvironment possibly diluting the effect that Rac1 deletion in all preosteoblasts might have. One further limitation was that we used Rac1^fl/fl^ as controls for Osx Rac1 after establishing that Osx^+/+^ had no effect on bone mineral density. The reason for choosing Rac1^fl/fl^ was that, in our opinion, littermate controls need to be used. The mating scheme providing 50% Osx Rac1 and 50% Rac1^fl/fl^ was therefore appropriate. In contrast, a scheme in which Osx^+/+^ would be generated in the same mating would include Osx Rac1^fl/+^ in both parents with a relatively low chance of getting the correct genotype and a littermate control in the same litter (Rac1^fl/fl^ 12.5%, Osx Rac1 12.5%, and Osx^+/+^ 12.5%). Finally, it is possible that other members of the Rho GTPase family can take over some but not all of the functions of Rac1 in osteoblasts. For this reason, all our conclusions are limited to Rac1 deletion.

In summary, we have shown that the absence of Rac1 in preosteoblasts diminishes their differentiation in vitro and function in vivo leading to lower bone mineral density. However, Rac1 is not required for PTH-mediated anabolic effects or for α4β1-integrin-mediated enhancement of osteoblast differentiation.

## 4. Methods

### 4.1. Mice

Homozygous Rac1 floxed mice were mated with mice carrying the osterix-1 promoter attached to Cre recombinase over two generations (Jackson Laboratory, Bar Harbor, Maine, USA) [42,43]. The osterix promoter contains a tetracycline responsive element and treatment with doxycycline of pregnant mice and after delivery leads to repression of Cre recombinase expression. Doxycycline was administered in drinking water (200 µg/mL in 5% sucrose solution). Drinking water was changed twice weekly. Studies in which doxycycline was stopped at the age of 2 weeks and bone mineral density measured at 3 weeks failed to show any changes in bone mineral density (Supplementary Figure S2). We, therefore, present only in vivo data from mice that did not receive any doxycycline after birth. For the isolation of newborn osteoblasts, the pregnant mice were not exposed to doxycycline. Mice were analyzed at the age of three weeks after calcein injections, which is laid down in the matrix and allows us to perform dynamic histomorphometry. Calcein in 0.9% NaCl was administered at 30 mg/kg 4 and 1 day prior to euthanasia [32]. For the study of the effect of PTH injection, bone mineral density (BMD) was measured at the age of 4 weeks in CT Rac1^fl/fl^ and Osx Rac1 mice under anesthesia with ketamine/xylazine. Mice were then divided into two CT Rac1^fl/fl^ groups and two Osx Rac1 groups based on the mean and the range of BMD measurements. One group in CT Rac1^fl/fl^ and one group in Osx Rac1 received daily vehicle injections and the other groups daily PTH injections at a dose of 80 μg/kg/day for 30 days. At the end of the experiment, mice were killed, and BMD measured. The changes in BMD are presented as percentages that represent the increase or decrease in bone mineral density of the second value based on the first value (for the first pair shown in Figure 4B: percentage change = (treated-baseline)/baseline). Human 1-34 PTH was used (Sigma-Aldrich, Taufkirchen, Germany). All animal studies were performed in accordance with regulations for animal welfare and approved by the responsible agency (Regierungspraesidium Karlsruhe, Land Baden-Wuerttemberg, Karlsruhe, Germany) under the following numbers: G-264/12, G-75/13, G-29/15, G-179/16 (The last two digits represent the year of approval).

### 4.2. Measurement of Bone Mineral Density

Bone mineral density was measured in the femur in vivo under anesthesia and ex vivo after fixation using peripheral quantitative computer tomography of the distal femur (Stratec, Pforzheim, Germany), with the reference line set at the growth plate and at a site located at 7.5% of the total length below the reference line. Total bone mineral density is shown in the figures. Data were analyzed as described using peel mode 20, and with the threshold set at 400 [44]. Bone mineral density was measured in 3-week old mice, except for the experiments using PTH, where the bone mineral density was measured at 4 weeks and 30 days later.

### 4.3. Bone Histomorphometry

Bones were fixed in 4% paraformaldehyde and embedded in polymethylmethacrylate, sections made and stained using Masson-Goldner. Photographs were obtained before and after Masson-Goldner staining for histomorphometry, which was performed as described [44]. Briefly, unstained 3-μm sections of the proximal tibia were used for dynamic histomorphometry and Masson-Goldner–stained sections for static histomorphometry. Histomorphometric analysis was performed according to the standards set forth by the ASBMR and used worldwide [45,46]. The primary spongiosa with a thickness of 150 μm was excluded and the cortical and trabecular bone in the area along the longitudinal axis of the tibia for a total length from the exclusion line of 1.5 mm was evaluated. The following parameters were examined: single and double-labeled surface (sLS, dLS), distance between labels, mineralizing surface (MS = dLS + ½sLS, mm), mineral apposition rate (MAR = distance between labels/4 days, μm/d), bone formation rate (BFR = [MS × MAR]/BS × 365/1000, mm/yr), bone surface (BS, mm), osteoid surface (OS, mm), osteoblast number (Ob.N.), osteoclast surface (Oc.S., mm), osteoclast number (Oc.N.), and osteoid thickness (O.Th., μm). Secondary parameters were BV/TV (%), OS/BS (%), MS/BS (%), Ob.N./BS (number/mm), Oc.N./BS (number/mm), Ob.S./Ob.N. (μm), Oc.S./Oc.N. (μm), Oc.S./BS (%), adjusted apposition rate (Aj.AR = MAR × MS/OS, μm/d), and mineralization lag time (Mlt = O.Th./Aj.AR, days).

### 4.4. Isolation and Mineralization of Osteoblasts, Inhibition, Stimulation, and Transfection

Newborn calvarial osteoblasts were isolated at the age of P1-3. Isolated calvariae were incubated with collagenase NB4 (Serva) and dispase (Gibco) and cultured in α-MEM + 10% FCS. Osteoblast mineralization was induced by adding medium containing β-glycerophosphate, vitamin C and dexamethasone thrice per week as described [12]. At the end of mineralization, wells were stained using the von Kossa stain and area quantified using ImageJ (Wayne Rasband, National Institutes of Health, Maryland, USA). An alkaline phosphatase activity assay was performed using a colorimetric method as described by Bessey, Lowry, and Brock except for the use of ZnCl_2_ in the substrate solution [47], and osteocalcin mRNA expression was determined using qPCR and adjusted to HPRT [6]. Parathyroid hormone was purchased (Human PTH 1-34, Sigma-Aldrich, Taufkirchen, Germany) and dissolved in 10 mM acetic acid and 0.1% BSA and added once to the medium at a final concentration of 5 nM. mRNA was collected 24 h later. Constructs for FN, FN-EDA and FN-EDB were based on the human plasma fibronectin cDNA clone (DKFZp686M04163) and a cDNA fibronectin clone containing the EDA and the EDB domain (DKFZp696O1166) which were prepared as described [12]. For expression in osteoblasts, the fibronectin cDNAs were cloned into the pmax cloning vector. Transfected osteoblasts were differentiated for 2–3 weeks and stained with von Kossa stain, used for mRNA expression analysis or for an alkaline phosphatase activity assay in the medium. The inhibitor used was EHT1864 (Tocris, Wiesbaden, Germany) at a final concentration of 5 μM added with each medium change.

### 4.5. RNA Analysis

RNA analysis was performed as described [48,49] and probes for osteocalcin #32, Rac1 #77, WISP-1 #76, HPRT #95 with primers as suggested by Roche universal probe library were used.

### 4.6. Western Blotting

SDS-PAGE (10%) was performed and Rac1 was detected using a rabbit polyclonal antibody #2465 (Cell Signaling Technology, Leiden, The Netherlands). GAPDH was used as a loading control #G9545 (Sigma-Aldrich, Taufkirchen, Germany). For analysis of ERK and AKT phosphorylation, cells were transfected using the TurboFect kit (Thermo Fisher Scientific, Rockford, Illinois, USA), left for 2 days in medium containing fibronectin-depleted FCS and harvested. The antibodies used were: pERK 1/2 (detecting phosphorylation at Thr202/Tyr185) #4376, ERK 1/2 #9102, pAKT (detecting phosphorylation at Ser473) #9271, AKT #9272 (All four antibodies from Cell Signaling Technology, Leiden, The Netherlands).

### 4.7. Statistical Analysis

Analyses were performed using SPSS (V24). Most data were compared using paired, unpaired *t*-tests or nonparametric tests. Analysis of variance (ANOVA) and repeated measures analysis of variance tests were used as appropriate. Whenever global probability was smaller than 5%, comparisons between pairs were performed as appropriate. Results are expressed as mean ± SEM.

## Supplementary Materials

The following are available online at https://www.mdpi.com/1422-0067/21/2/385/s1, Supplementary Data: Uncropped Western blots and cropped Western blots shown side by side, Supplementary Figure S1: Dose response to a chemical inhibitor of Rac1 in osteoblasts, Supplementary Figure S2: Bone mineral density after one week repression of Osx-cre, Supplementary Figure S3: Bone histomorphometry changes in Osx Rac1 mice compared to CT Rac1^fl/fl^.
