# Supplementary material for: The Rho GTPase RAC1 in Osteoblasts Controls Their Function
Source: Int J Mol Sci. 2020 Jan 8;21(2):385. doi: 10.3390/ijms21020385 (PMC7014472; doi:10.3390/ijms21020385)
Supplement: Supplementary file 1 [file ijms-21-00385-s001.pdf]

Supplementary Figure S1

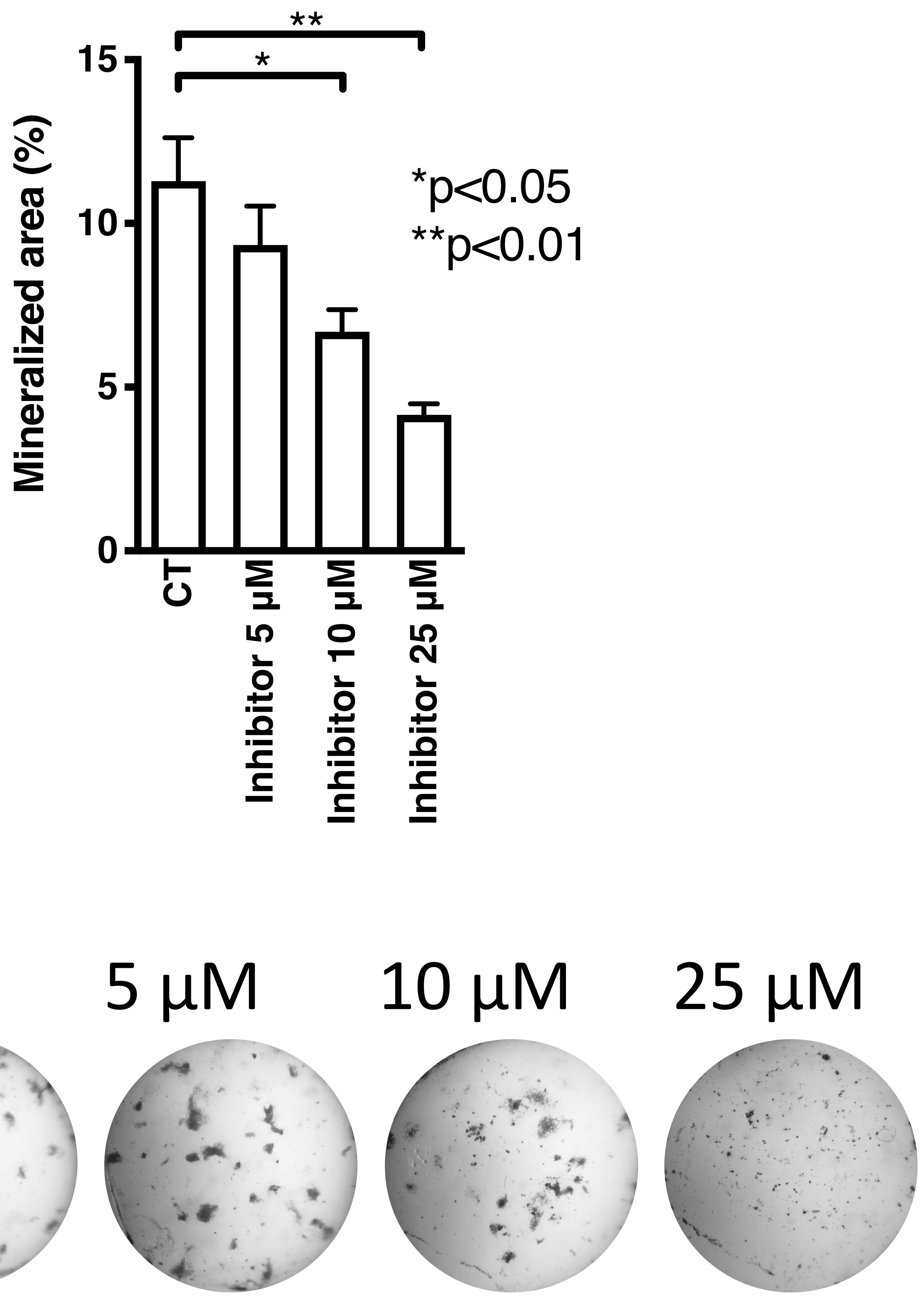

Supplementary Figure S1

Dose response to a chemical inhibitor of Rac1 in osteoblasts

Using a chemical inhibitor of Rac1 (EHT1864) added with each medium change to osteoblasts in differentiation media at a final concentration of 5, 10 and 25 µM suppressed differentiation of newborn calvarial osteoblasts as evidenced by suppressed nodule formation in cultures stained with von kossa compared to control cells treated with PBS containing DMSO at a concentration similar to the highest concentration of the inhibitor. N=5/4/4/3. The values were compared between all groups using one-way ANOVA. This was significant at $p<0.005$ allowing further analysis. The treated groups were compared to the control (CT) using unpaired t-test.

Supplementary Figure S2

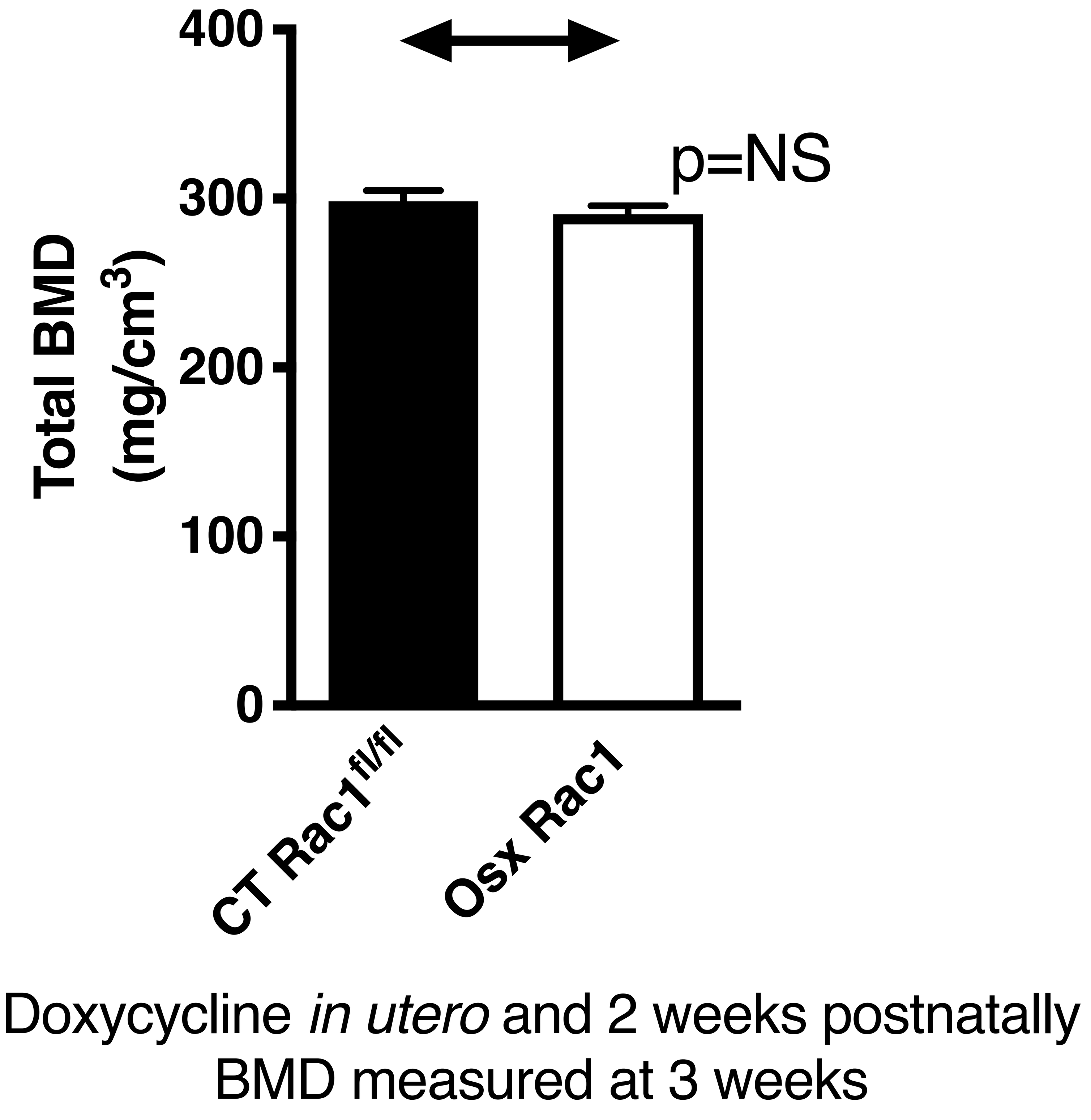

Supplementary Figure S2

Bone mineral density after one week repression of Osx-cre

Repression of Osx-Cre expression prenatally and for two weeks after birth by administering doxycycline to the mothers and stopping doxycycline for 1 week before death is not enough to affect bone mineral density measurement. N=21/17. Values were compared by unpaired t-test.

Supplementry Figure S3

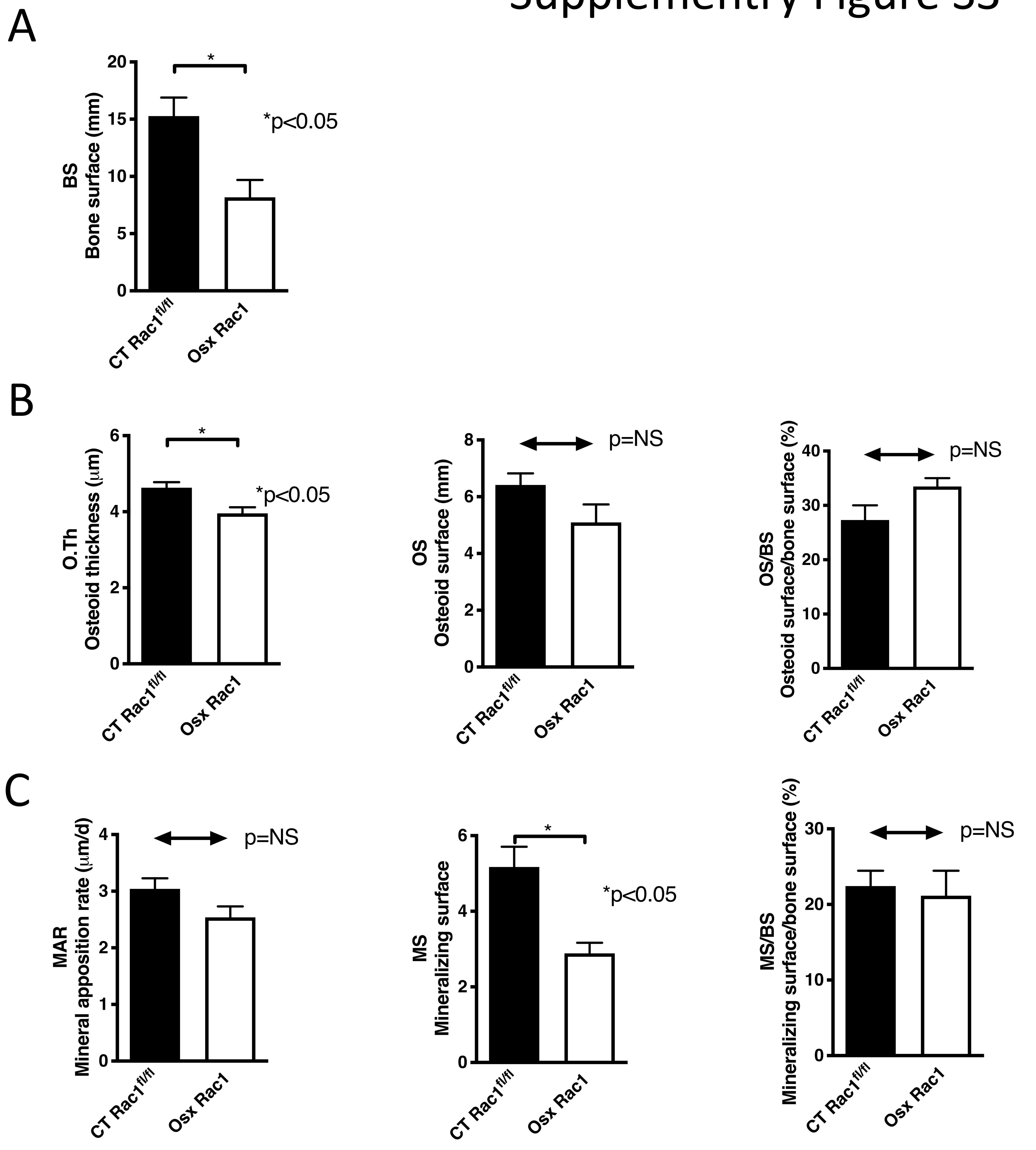

Supplementary Figure S3

Bone histomorphometry changes in Osx Rac1 mice compared to CT $Rac1^{fl/fl}$. The data complement those presented in Figure 3 and include the same mice and the same number of replicates. (A) Deletion of Rac1 in preosteoblasts diminishes bone surface to about 50% compared to control littermates. (B) Osteoid thickness (O.Th.: matrix laid down by the osteoblasts, Osteoid surface (OS: bone surface covered by unmineralized osteoid) and OS/BS: osteoid surface in relation to bone surface showed that in Osx Rac1 mice, less osteoid was laid down by the osteoblasts. (C) Mineral apposition rate (MAR: which represents the speed at which osteoid is mineralized) was not slower, but mineralizing surface (MS: represents the bone surface that is mineralized; i.e. calcein labeled) was diminished. MS/BS which represents the mineralizing surface in relation to bone surface (BS) was no longer significantly diminished. N=10/5. The values were compared between each two groups in all subpanels using t-test.

Supplementary Data

A. Figure 1B

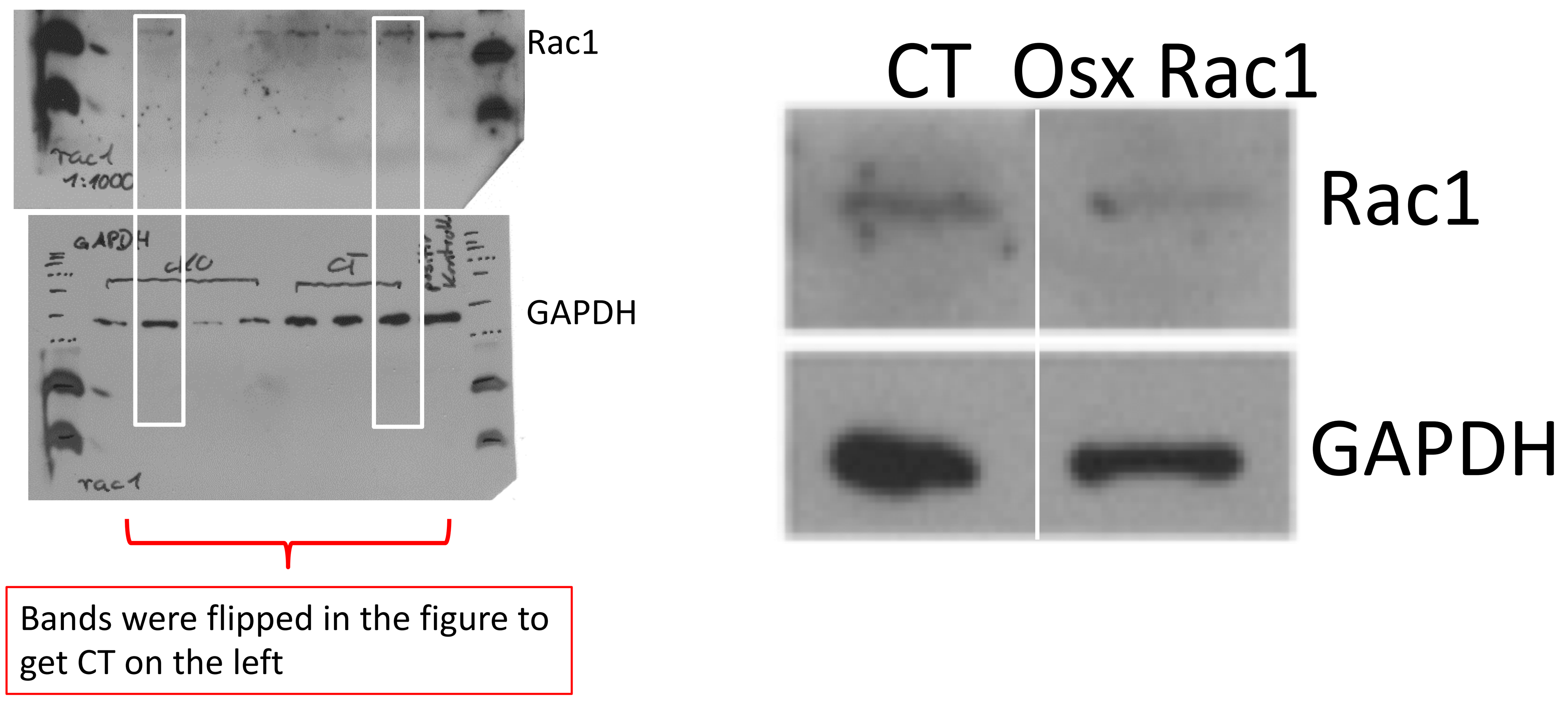

B. Figure 5A

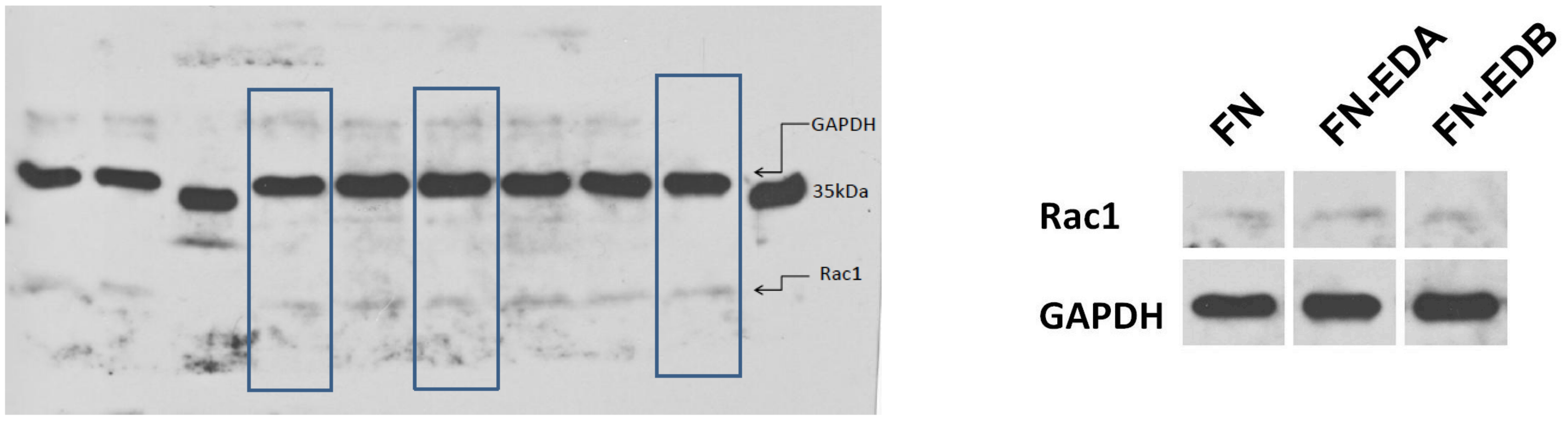

C. Figure 5C

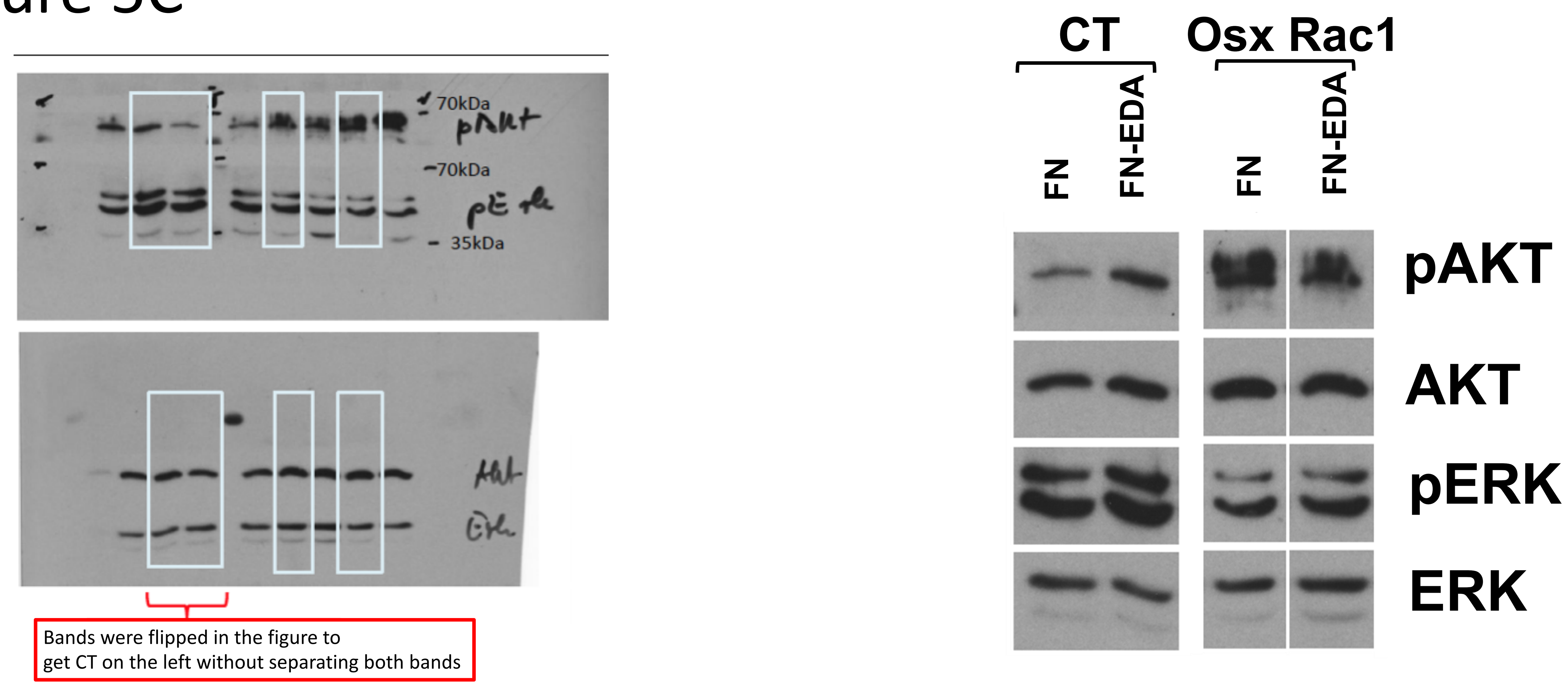

Supplementary Data

Uncropped Western blots and cropped Western blots shown side by side

(A) The uncropped Western blots for Rac1 in CT Rac1$^{fl/fl}$ and Osx Rac1 osteoblasts are shown on the left. On the right, the panel from Figure 1B is replicated. (B) The uncropped Western blots for Rac1 after transfection with the three fibronectin constructs is shown on the left. On the right, the panel from Figure 5A is replicated. (C) The uncropped Western blots for pAKT, AKT, pERK, and ERK are shown on the left. On the right, the panel from Figure 5C is replicated.
